# Dissemination in Extension: Health Specialists’ Information Sources and Channels for Health Promotion Programming

**DOI:** 10.3390/ijerph192416673

**Published:** 2022-12-12

**Authors:** Thomas E. Strayer, Laura E. Balis, NithyaPriya S. Ramalingam, Samantha M. Harden

**Affiliations:** 1 Center for Quality Aging, Vanderbilt University Medical Center, Nashville, TN 37208, USA; 2 Human Nutrition, Food, and Exercise, Virginia Tech, Blacksburg, VA 24060, USA; 3 Gretchen Swanson Center for Nutrition, Omaha, NE 68154, USA; 4 Office of Postdoctoral Affairs, Oregon Health & Science University, Portland, OR 97239, USA

**Keywords:** Cooperative Extension, health promotion, dissemination, diffusion of innovations, mixed-methods, translational science

## Abstract

In the National Cooperative Extension System (herein: Extension), state-level specialists serve as key intermediaries between research, educators, and the community members they serve. There is a need to understand information seeking and sharing practices (i.e., dissemination) among specialists to increase the adoption of evidence-based health promotion programs. Specialists (*N* = 94) across 47 states were identified and invited to participate in this mixed methods study. A one-way ANOVA with Bonferroni corrections was used to analyze survey data. Data collected through semi-structured interviews were analyzed using an immersion crystallization approach. Forty-seven health specialists completed the survey representing 31 eligible states (65%) and were predominately female (89%), Caucasian (70%), had a doctorate (62%), and were employed within Extension for 10.2 + 9.7 years. The information sources used most frequently were academic journals and other specialists, and most used email and online meetings to communicate. Qualitative findings support the use of other specialists as a primary source of information and indicate specialists’ desire for an on-demand, bi-directional, online national repository of Extension programs. This repository would facilitate the dissemination of evidence-based programming across the system and reduce program duplication as well as information burden on county-based educators.

## 1. Introduction

The land-grant university Cooperative Extension System (Extension) is a federally funded organization formed in 1914 with the mission to “bring the university to the people” [1,2]. This community-centric system translates research to practice through delivering health promotion programs covering a range of topics, from health literacy to chronic disease prevention to food safety [3]. Extension health educators (county-based, total staffing levels vary by state) serve as a workforce to identify needs, match programming to meet those needs, and then adopt and deliver best-fit programs to millions of Americans [4]. These community-based health educators primarily receive information on health promotion interventions through other health educators and from Extension specialists state-level experts who support health educators through training and technical assistance [5,6,7,8]. Thus, specialists are key to the translational science process, as they determine which research reaches practitioners and communities [9].

While Extension is available in every state and territory, state-level operations are distinctively different, and there is no centralized entity (e.g., governing body or leadership) to link specialists to each other. There are committees, such as the National Institute of Food and Agriculture’s (NIFA) Nutrition and Health Committee for Planning and Guidance [10], and learning collaboratives through eXtension (a foundation supporting collaborative Extension work). However, communication across these committees and collaboratives has been limited, and program gaps and duplication remain a problem for national, collective impact. For example, there are 17 unique older adult physical activity programs and 14 unique walking promotion programs delivered across the country, and many of these programs are not evidence-based [11,12].

Taken together, there is need to understand information seeking and sharing practices among specialists. In other words, how information actively travels, i.e., dissemination, is key to scaling-out evidence-based programming to new settings and populations [13]. Dissemination can be defined as three constructs: sources of information, channels of communication in which information can be delivered to target audiences, and target audiences’ characteristics [14]. Sources are where the information is located (e.g., colleagues, academic journals) while channels are how the information is communicated (e.g., e-mail) [8]. Priority audience characteristics impact this process, and the information conveyed in dissemination should match the audience’s (rather than only the developer’s) aims to enhance program adoption and delivery [15,16,17].

Once the sources, channels, and target audience characteristics are defined, a dissemination strategy can be developed. Dissemination strategies focus on distributing evidence-based interventions and materials to defined audiences through specific channels [18]. For example, this could include distributing customized messages through newsletters to decision makers to impact evidence-based intervention knowledge adoption [18]. To select and tailor relevant dissemination strategies, an understanding of contextual factors—including individual characteristics and organizational culture—is necessary [19,20]. This information can be used to scale out a dissemination strategy to other community settings (e.g., state and local health departments) [21,22,23] through a rapid process of identifying common and unique contextual factors across settings [24,25]. Thus, the aims of the study reported here were to identify (a) information sources and channels used by specialists to obtain programming information, and the channels of communication used between educators and specialists; and (b) specialists’ perceptions of an intervention to improve the dissemination of evidence-based health promotion programming to health educators.

## 2. Materials and Methods

### 2.1. Framework

The literature within the field of dissemination science acknowledges a barrier in choosing a framework from the over 100 frameworks and models that currently exist [26]. The most used theory within dissemination research is the Diffusions of Innovations (DOI) Theory [27,28]. DOI describes the characteristics of innovations, adopting and distributing audience, and organizations that may speed or impede translation into practice (especially in national systems such as Extension) [29,30]. However, DOI does not explicitly include how information delivery occurs, and thus a gap exists in learning about the information sources and channels as well as the adoption and distribution of evidence-based practices used by Extension specialists. To fill this gap, the Enterprise Planning theory has been used to augment DOI by aiding in the identification of communication channels within organizations [21,31]. Enterprise planning includes internal resources and methods, and has been primarily used in industry settings to translate policies and practices across and within organizations [21,31].

### 2.2. Design

A sequential explanatory mixed-methods approach (the quantitative portion occurring prior to the qualitative stage) was used for this study [32]. Mixed-methods research is useful for describing complex phenomena like dissemination, and was necessary to determine how Extension specialists interpret key sources of intervention information (e.g., academia or dissemination tools) and allow for the data to be in reported in unique categories of meaning created by specialists [32,33].

### 2.3. Participants

As there is no federal list of Extension state specialists who support health promotion, the research team developed a contact list in previous work [8]. This list was composed from publicly available data from land-grant universities and membership data found on the NIFA Nutrition and Health Committee for Planning and Guidance. Extension health state specialists typically have a terminal doctoral degree (e.g., DrPH, Ph.D., EdD) and focus on areas within health promotion such as nutrition, physical activity, or food safety. While staffing structures and funding availability vary, states typically hire one specialist per focus area. Specialists across the nation have different degrees, qualifications, responsibilities, and titles as dictated by their state system [10,34]. To be eligible for this study, the specialists must have been currently employed in a role as a health specialist within Cooperative Extension. Additionally, specialists were incentivized with the chance to win a $50 gift card for participating in the survey or interview portions of this study.

### 2.4. Survey

The survey was designed to explore the following questions:

1. *Which information channels and sources are used to influence the intervention adoption-decision making process for Extension health specialists and channels used for educator communication?* Which sources (e.g., journals, other specialists, etc.) and channels of communication (e.g., email, phone, face-to-face, etc.) are used most often (1—Never; 5—Most often use) to seek intervention information? Response options were informed by previous literature on Extension [5,7,8,14,31].
2. *Which channels communication and frequency of communication do specialists utilize with educators?* Which sources (e.g., journals, other specialists, etc.) and methods of communication (e.g., email, phone, face-to-face, etc.) are used most often (1—Never; 5—Most often use) to seek intervention information? Response options were informed by information sources, channels, and previous literature on Extension [5,7,8,14,31].
3. *What are the specialists’ perceptions of a dissemination strategy and interest level surrounding dissemination in Extension?* This question explored the demand of a dissemination strategy (e.g., How useful do you believe a dissemination (the active targeting of information delivery) intervention would be that actively distributed new evidence-based practices to you, specialists, that you could use to distribute to Extension health educators? (1 = Not at all Useful and 5 = Extremely Useful)
4. *Demographic variables* were assessed based on standard variables in methodology literature [35,36] and previous work [37,38,39] and included race, ethnicity, sex, age, state of employment, official role title within Extension, duration of employment within Extension, and educational degree and field.

#### Survey Analysis

The survey consisted of Likert-scale and rank-ordered response. These questions were analyzed by a One-way ANOVA, and when significant were followed-up with a Bonferroni Post hoc test. Parametric tests were performed for this analysis based on the recent justifications that ordinal Likert-based responses were reliable and accurate [40]. Stacked bar charts were chosen to convey the sample’s distribution across response rankings. The analysis of the survey data was used to inform the semi-structured interviews.

### 2.5. Semi-Structured Interviews

Semi-structured interviews with specialists were conducted within two months after survey conclusion. Health specialists (*N* = 12) were selected to participate through purposive, multi-site sampling [41]. This sample size included three Specialists in each of the four national districts (Northeast, North Central, South, and West) as was deemed likely to achieve saturation [42]. Two specialists agreed to interview in the Northeast district and only two were able to complete interviews in the West district, for a total of ten interviews (83% response rate). The process was identical to that used in previous work related to health educator processes [8]. The survey portion of this study included the consent to be contacted for interviews by participants voluntarily providing contact information to complete the interview. Additionally, informed consent was received at the start of the interview. Our study was determined to be Western Institutional Review Board (WIRB) exempt; we based our practices on the previous educator study IRB protocols [8].

#### Semi-Structured Interview Analysis

An immersion crystallization approach was used to analyze interview data to maintain the interconnectedness of the data [43]. This process involved the researchers being immersed in the data and iteratively going through the themes and process of the information network within Extension [43]. Trained researchers independently coded meaning units [33], and a critical friend (the senior researcher) assisted in reconciling any coding or meaning unit discrepancies between independent coders [33,44]. Meaning units can be defined as portion of content that serves as the foundation for the development of coding and can appear as a word, sentence, or paragraph but contain a single code [33]. Inter-rater reliability techniques were applied to ensure reliable analyses and resolve conflicts, and was established by having each member of the research team (TS and NR) independently code one participant’s interview and then compare and reconcile results. Inter-rater reliability was initially greater than 85%. Following reconciliation, the remaining meaning units of a single interview were coded. The second round of inter-rater reliability was 92%. The remaining meaning units were then independently coded by members of the research team.

## 3. Results

### 3.1. Sample

Extension health state specialists were identified in 45 states and invited to complete a Qualtrics survey. Participants were sent an initial invitation email and follow-up emails sent three days apart for the four-week span. A total of 62 specialists started the survey, but only 47 (77%) of the responses were completed and met eligibility criteria. A response rate of 50% was achieved from the original 94 identified specialists with responses from 30 (69%) of the identified states. Table 1 provides additional demographic information about the specialist population for this study. Appropriate testing (ANOVA, *t*-test) on demographics did not yield a statistical difference between those that completed an interview versus those that responded to the survey. While no national database of specialists exists, the demographics of participating specialists are similar to those reported in other research [45].

### 3.2. Quantitative Results

#### 3.2.1. Specialists’ Information Sources and Channels for Programming Information

**Figure 1:**
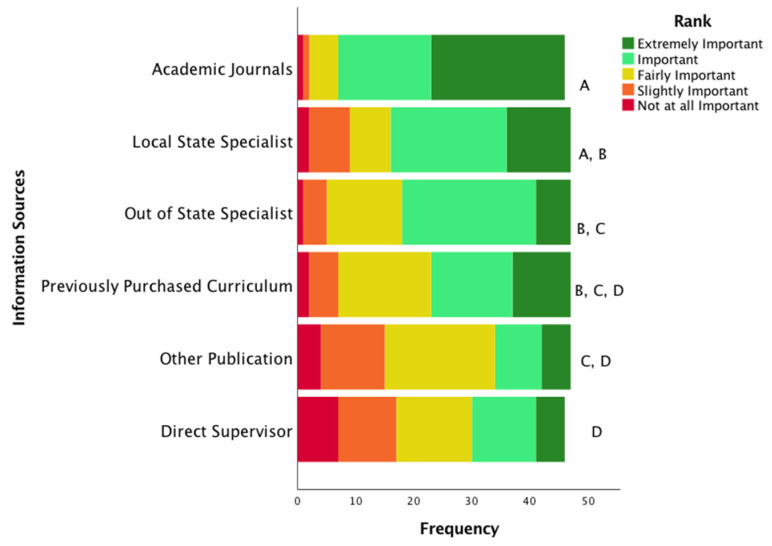
Specialists’ Information Sources. These data are reported in a frequency format with color-coded stacked bars representing the frequency of the sample that valued each information source. Information sources are listed in order of significance. Lettering at the end of the bars were indicates statistical differences between each source. That is, each letter indicates the presence of a statistically significant difference between that source and any differently lettered sources. Identical lettering indicates that there is no statistical difference (*p* > 0.05) between sources. For example, “Previously Purchased Curriculum,” lettered “BCD,” is only statistically different than “Academic Journals,” which is labeled as “A”.

Figure 1 shows the frequency of each response from right to left of “not at all likely” to “extremely likely.” Larger bar sections represent greater importance of that information source. The sources were also ordered from top to bottom based on the mean score of the Likert responses from highest to lowest. ANOVA indicated that there was a significant difference among the relationships of the information sources (*p* < 0.05). Using a Bonferroni post hoc test, it was determined that the significance was between academic journals and all other information sources except when compared to using other state specialists. Additionally, local specialists were also used more often than other publications and direct supervisors (*p* ≤ 0.05). The direct supervisor response was the least reported source for information about a health promotion intervention with other publications being the second lowest reported source.

**Figure 2:**
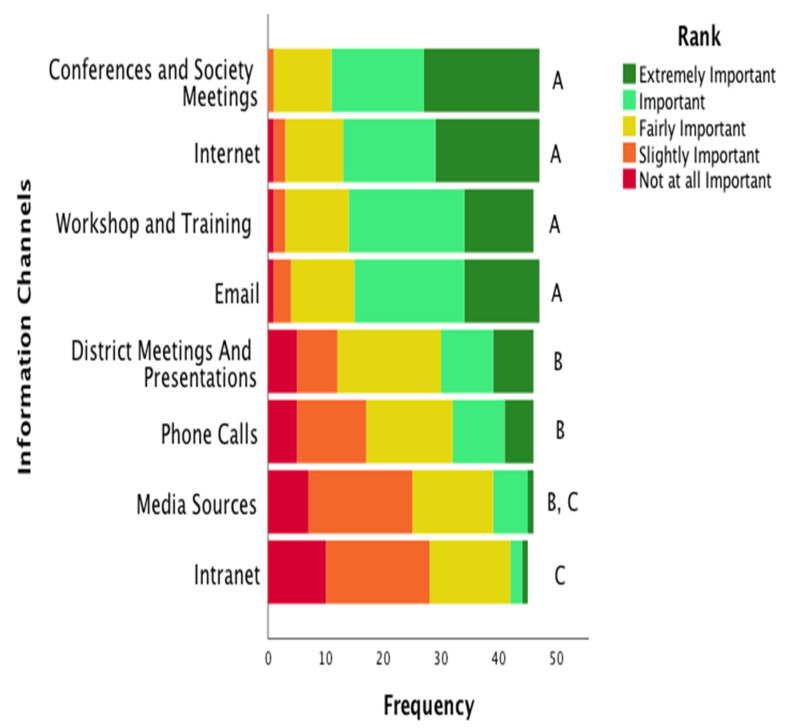
Specialists’ Information Channels. These data are reported in a frequency format with color-coded stacked bars representing the frequency of the sample that valued each information channel. Information channels are listed in order of significance. Lettering at the end of the bars were indicates statistical differences between each channel. That is, each letter indicates the presence of a statistically significant difference between that channel and any differently lettered channel. Identical lettering indicates that there is no statistical difference (*p* > 0.05) between channels.

The channels of information communication consisted of the responses in Figure 2. The most important channel of communication for specialists to receive information about health promotion interventions were conferences and society meetings as well as the internet. These channels were followed by workshops, trainings and email. Phone calls, district meetings and presentations, media sources, and the intranet were reported as the least likely used communication channels and were statistically different from the remaining communication channels.

#### 3.2.2. Specialists’ Frequency and Channels for Educator Communication

**Figure 3:**
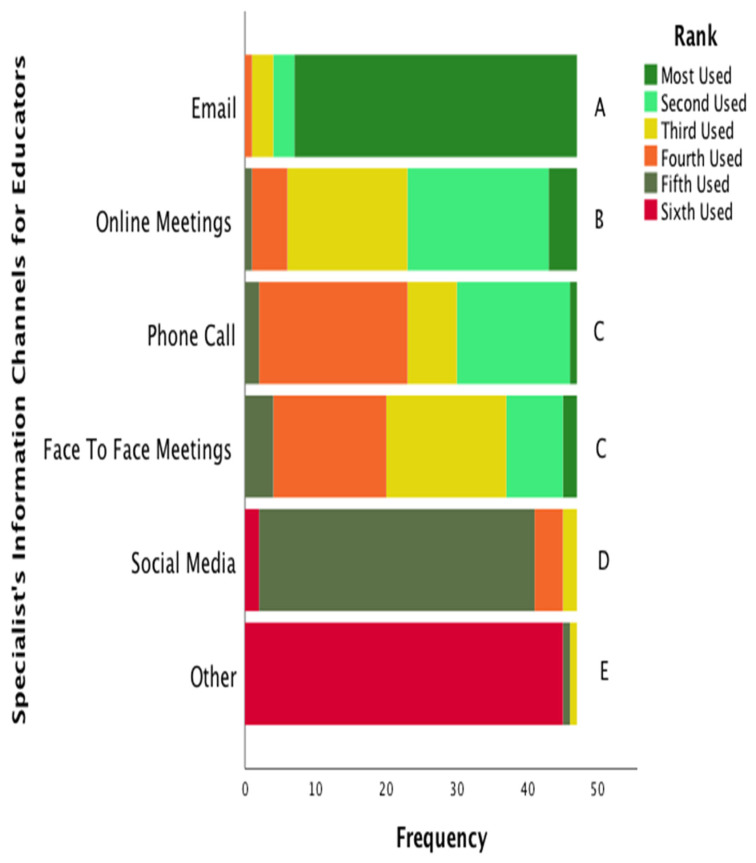
Specialists’ Information Channels Used for Educator Communication. These data are reported in a frequency format with color-coded stacked bars representing the frequency of the sample that valued each information channel. Information channels are listed in order of significance. Lettering at the end of the bars were indicates statistical differences between each channel. That is, each letter indicates the presence of a statistically significant difference between that channel and any differently lettered channel. Identical lettering indicates that there is no statistical difference (*p* > 0.05) between channels.

Twenty-seven specialists (57%) reported communicating in some way with educators one to three times a month or less. A majority of specialists (*n* = 31, 66%) reported communicating with health educators less than once a month. Figure 3 represents the communication channels ranked in order of use.

### 3.3. Qualitative Results

A total of three themes were developed: Specialist Information Sources and Channels, Specialist Communication with Educators, and Initial Perceptions of a Dissemination Strategy. Within each theme, sub-themes were identified. Within each sub-theme, additional descriptions are included to elucidate details (e.g., frequency of use, specific information channels, and potential improvements to information sources) Frequencies are included as the number of specialists who contributed meaning units to each sub-theme and sub-theme description. See Table 2. Below, each theme is reported with the most common sub-themes and a summary of the sub-theme descriptions.

#### 3.3.1. Specialist Information Sources and Channels

Within the theme of Specialist Information Sources and Channels, specialists reported that they frequently used academic journals. Few specialists (*n* = 2) shared the channels by which they access journal articles, mentioning that they used the Internet, such as Google Scholar and specific journal websites, and that many publications sent updates via email. The direction of dissemination was reported as bi-directional in that specialists aimed to understand what other specialists were reporting as well as reporting their findings via peer reviewed journal articles. As for perceptions, most specialists (80%) stated that while academic journals were trustworthy for evidence-based information, they were not incredibly useful for finding specific programming to adopt and deliver. For example: “…if you were wanting to find an intervention just simply through a journal, you know, what would you search for?”.

A second prominent information source was other specialists. The channels in which specialists communicated with other specialists included conferences, meetings, email, and phone calls. Conferences and meetings for specialist were the most mentioned channels for communicating with other specialists: “When I’m at conferences and things like that, I generally lean on them [other specialists] … to hear more about the interventions that they use.” Specialists reported using a mixture of in- and out-of-state specialists to learn about new programming information. The majority of specialists interviewed, with the exception of one, believed that other specialists were trustworthy sources of evidence-based health promotion interventions and related programming information. The one specialist who reported an absence of trust mentioned that specialists lacked a uniformed qualification in health promotion programming (i.e., they have varying degrees and specialties).

The third prominent information source was the use of government sponsored organizations for programming information. The information channel used for reaching these resources was the internet, such as online repositories or Extension websites. Specialists mentioned both actively contributing to these resources and also receiving information from these sources, similar to the bi-directional process used with academic journals. The sources consisted of resources such as the CDC, Extension websites, USDA, and the NIH. Specialists reported that each organization mentioned was a trustworthy source of information and often had non-branded resources easy to integrate into Extension practice. While these resources are considered trustworthy, it was mentioned that they would benefit from more frequent updating.

#### 3.3.2. Specialists Communication with Educators

The second theme was around the process of specialists’ communication with educators; key channels were email, trainings, and web-based tools. Specialists primarily communicated with educators via email, with content focused on community needs and how to address them. Specialists reported that the frequency of communication with educators was on an as needed basis, typically daily to weekly, dependent on current state needs. Often, emails were sent in a listserv format to reach multiple educators, as the number of educators per state ranges from eight to 150. Specialists valued the importance of having an open communication channel with educators, “I really see my… one of my main rules in my job is to support the agents (educators) and so… in doing so you need to communicate”.

While training was not an original option in the study as a form of communication, specialists often mentioned that they get programming information to educators through training. The content of the trainings was Extension-approved programming, and the frequency was intervention dependent. Trainings were described a direct and purposive way for specialists to relay information to educators, with a preference for face-to-face communication. Finally, web-based tools such as WebEx, Zoom, or Dropbox were mentioned as ways to deliver large amount of information to multiple targets quickly. Specialists reported that educators appreciated this means of communication as it was easier to facilitate meetings.

#### 3.3.3. Initial Perceptions of a Dissemination Strategy

Specialists believed other specialists were the most reliable resource to learn about Extension programming nationwide, including intervention-specific information and Extension intervention information. The information channels mentioned were email and an online repository with one mention of phone and webinar. Email was the most commonly mentioned information channel, but specialists were also the most cautious with this method, particularly with email burden (e.g., “I think that… our agents (educators) are overwhelmed with email…”) as specialists believed that too many emails would lead to the reduced attention to the information that specialists disseminate, “our agents (educators) are overwhelmed with email. Oh I don’t… my specialist emails would not be…business. Who wants another damn email?” Finally, interviewed specialists described a two-level dissemination pathway: (1) specialist to specialist and then (2) specialist to educators.

## 4. Discussion

This work aimed to gain an understanding of the information channels and sources used by state-level specialists to learn about health promotion information, how this information is communicated from specialists to community-based health educators, and the initial perceptions of specialists regarding a potential dissemination strategy to improve program translation in Extension nationally. Ultimately, such an intervention could be scaled out to additional settings beyond Extension. Survey results indicated that specialists use academic journals and other specialists to learn about health promotion programming information. In the process of finding programming information, academic journals were used mainly for generic evidence-based information and not specific health promotion programming. It became clear that other specialists were key sources for programming information and were reached through conferences and meetings. In previous literature, specialists were noted as being valuable sources of information for Extension educators and technological means of communication were used for information channels [5,6,7,8]. This study found similar results in that specialists valued information from other specialists, but conferences as the main channel that specialists used to communicate was a new finding. Therefore, while conferences and travel are often seen as a privilege, they are a key to dissemination and should be prioritized by the land-grant system (e.g., through provision of time and money).

With regard to frequency and channels for communicating with educators, specialists self-reported communicating with educators weekly or less as a group, but often spoke to individual educators daily or weekly. Specialists reported that their infrequency of contact was underscored by the desire to not overburden the already over worked educators [46,47]. However, this is a potential place for a dissemination strategy, as health educators desire more communication from specialists on evidence-based health promotion interventions [8]. One method to improve specialist and educator communication could be through an active approach of specialists reaching out through listservs or state meetings to better inform educators of specialists’ roles and availability. Taking a more active role in this relationship may promote regular communication rather than spontaneous specialist-educator interactions.

Specialists reported the ideal dissemination strategy would include information available on an as-needed basis, such as a directory of specialists and their expertise as well as an online repository of Extension-tested interventions. Previously, repositories have been discussed as resources for use within Extension, specifically for evidence-based intervention components [12,48]. At the time of this study, no Extension program repository existed. A repository was launched in 2021 (see extension.org/registry); however, this tool does not require entries to provide any evidence on needed Rogerian characteristics (e.g., adaptability or ease of delivery of the intervention [30]; or empirical evidence of the intervention’s effectiveness and overall public health impact. Additionally, further research on the way information appears in such a repository must represent both specialists’ and educators’ preferences to maximize the effective uptake and delivery of evidence-based health promotion programming [49]. The online repository may be the best channel to let specialists provide information as the need arises, reducing the burden of over-information. In fact, a specialist stated, “I think some sorta repository data base, evidence based, evidence informed system, you know, like the Stanford data base for mental health extension…could be used”, implying that these repositories may aid in the facilitation of programming translation at a national scale.

This work is not without limitations. There was a lack of diversity in the specialists who participated in this study, which reflects a documented challenge within the Extension system [50]. Nationwide specialists’ demographic data on are not readily available and as such it is difficult to assume that the data in this work are generalizable or transferable to Extension as a whole. The results of this study are also limited by response bias and by the limitations of a semi-structured interview. Semi-structured interviews make each interview unique and allow for a variety of paths to be followed, thus the same topics were not necessarily explored in each interview. To represent this, we included measures of our qualitative data table to show the representativeness of the topics reported.

## 5. Conclusions

This study contributes to bridge the gap between the development of health promotion interventions and the eventual delivery of these interventions by the national Cooperative Extension System. The results provide implications for policy, practice, and research—in Extension and beyond. This includes the importance of scientific conferences for evidence-based program dissemination, and the use of listservs or state meetings for regular communication. As well, the results suggest that the future of information dissemination might be through a specialist-informed repository of online information that can be used to distribute health promotion interventions based on educator demand. As well, this repository could be used beyond the Extension system, as sharing resources with partner organizations is part of Extension’s mission [3,51,52,53]. Overall, the successful development of a dissemination strategy that utilizes desired sources and channels of information can impact the successful program translation of evidence-based programs, reduce programming duplication, and improve health outcomes for Americans.

## Figures and Tables

**Table 1:** Extension Specialist Demographics.

Demographics Variable	Survey Respondents (*N* = 47)	Interview Respondents (*N* = 10)
Gender		
Male *n* (%)	4 (9)	2 (20)
Female *n* (%)	42 (89)	8 (80)
Other *n* (%)	1 (2)	0 (0)
Age Mean (STD)	46.9 (±13.4)	40.1 (±12.9)
Spanish, Hispanic, or Latino		
Yes	3 (7)	0 (0)
No	44 (93)	9 (90)
Race		
White or Caucasian	33 (70)	10 (100)
Black or African American	7 (15)	0 (0)
Asian	1 (2)	0 (0)
Other	5 (11)	0 (0)
Percentage of time spent (Mean Percent) (STD)		
Research	21 (±19)	23 (±20)
Extension	68 (±30)	68 (±31)
Teaching	19 (±15)	21 (±15)
Duration as a Specialist in Cooperative Extension Mean (STD)	10.19 (9.69) years	7.86 (±9.5)
Highest level of Education *n* (%)		
Master’s degree (course option)	11 (23)	3 (30)
Master’s degree (Thesis Option)	6 (13)	1 (10)
Doctorate degree	29 (62)	6 (60)

**Table 2:** Specialist Interview Qualitative Results.

Theme	Sub-Theme (*n* = Number of Specialists Contributing Meaning Units)	Sub-Theme Descriptions (*n* = Number of Specialists Contributing Meaning Units)
**Specialist Information Sources and Channels**	**Academic Journals (10)**	**Frequency:** Use as needed (8) **Information Channel:** Internet (website/google scholar) (2) **Direction of Dissemination:** Bi-directional (8) **Perceptions:** Trustworthy source of information (9), For evidence-based information, not programming-specific (8), Journals [17 mentioned] (8) **Suggestions for Improvement:** More details in intervention methodology (1), Prefer journal article be supplemental to intervention information (1)
	**Specialist (10)**	**Frequency:** Use as needed (6), Rarely or infrequent (2) **Information Channel:** Conferences (7), Meetings (6), Email (3), Phone calls (2) **Direction of Dissemination:** Bi-directional (3), Actively reaches out (1) **Perceptions:** Use in-state specialist (6), Use out-of-state specialist (6), Trustworthy source of information (8), Not a trustworthy source of information (1), Good information source for programming information (6) **Suggestions for Improvement:** Programming can be costly coming from other specialist (1), Specialist should be more uniform in qualifications (1)
	**Government Organizations (10)**	**Frequency:** Use as needed (10) **Information Channel:** Internet (websites or online repositories) (10) **Direction of Dissemination:** Bi-directional (5) **Perceptions:** Use a variety of sources such as: CDC [Centers for Disease Control and Prevention] (7), Extension (8), USDA [United States Department of Agriculture] (4), eXtension (3), NIH [National Institutes of Health] (3), Federal Trade Commission (1), Public Health Department (1), Center TRT [Training and Research Translation] (1), Use trustworthy sources of Information (8), Non-branded resource (2) **Suggestions for Improvement:** Programming can be costly coming from other specialist (1), Specialist should be more uniform in qualifications (1)
	**Non-Profit Organizations (3)**	**Perceptions:** American Diabetes Association (2), American Heart Association (2), Dairy Council (1)
	**Private Organizations (1)**	**Perceptions:** Gatorade Sports Science Institute (1)
**Specialist Communication with Educators**	**Email (10)**	**Content:** Emails contain information on community needs or addressing community needs (10) **Frequency:** Daily-weekly (10), Listserv (as needed or scheduled updates) (7) **Direction of dissemination:** Bi-directional (10) **Rationale:** Most common channel to communicate with multiple educators (7)
	**Training (8)**	**Content:** Extension-approved programming for delivery (8) **Dose:** Intervention dependent (8) **Information channel:** In-service trainings/workshop (8) ○ Face-to-face (8) Webinar trainings (4) **Direction of dissemination:** Specialist training educators (8) **Rationale:** Specialist believe that training improves program adoption and fidelity (1), Needs a funding mechanism (1)
	**Web-based Tool (WebEx/Zoom/Dropbox) (5)**	**Content:** Statewide initiative information/health topics in communities/intervention information (4) **Frequency:** Monthly and based on health topics and intervention needs (4) **Direction of dissemination:** Specialist to educator (5) **Rationale:** Educators requested this channel of communication as it is convenient and easier to facilitate statewide meetings (3)
	**Social Media (5)**	**Rationale:** Specialists often state that social media was not a tool to be used to communicate with educators (5)
	**Additional Communication Information(Unique Insight provided by Specialist) (5)**	Communication may be a barrier that contributes to the underutilization of specialists (1) Believes educators can be more productive with more contact with specialist (1) Communication with educators is important especially to ensure educators have the most up to date intervention information (1) Personal relationships matter in this (specialist) position as they help improve program fidelity (1) Attention to emails is short so emphasizes not burdening educators (1) Mentions that communication should be as needed with educators and that amount is not a measure of success (1)
	**Phone (3)**	**Content:** Community need and intervention information (3) **Frequency:** Episodic (1), as needed (2) **Direction of dissemination:** Bi-directional (3) **Rationale:** Informal means of communication (1), Can use conference calls for multiple individuals, specialist also believes talking improves personal relationships increasing intervention success (2)
**Dissemination Strategy**	**Specialists (10)**	**Dissemination Content:** Intervention-specific information (9), Extension intervention information (7) **Frequency:** As needed for intervention information, updates, etc. (5), Monthly (1), Quarterly (2), Systematic approach to dissemination (1) **Information Channel:** Email (8), online programming repository (2), Phone (1), Webinar (1) **Direction of Dissemination:** Specialist to educators (9), Specialist to specialist then to educators (3)

## Data Availability

The data presented in this study are available on request from the corresponding author.

## References

[1] Rasmussen W.D. (2002). Taking the University to the People: Seventy-Five Years of Cooperative Extension.

[2] U.S. Department of Agriculture Extension https://nifa.usda.gov/extension.

[3] Braun B., Bruns K., Cronk L., Kirk Fox L., Koukel S., Le Menestrel S., Monroe Lord L., Reeves C., Rennekamp R., Rice C. (2014). Cooperative Extension’s National Framework for Health and Wellness.

[4] Battelle (2015). 2015 Battelle Study: Analysis of the Value of Family & Consumer Sciences Extension in the North Central Region.

[5] Bailey N., Hill A., Arnold S. (2014). Information-Seeking Practices of County Extension Agents. J. Ext..

[6] Mastel K.L. (2014). Extending Our Reach: Surveying, Analyzing, and Planning Outreach to Extension Staff. J. Agric. Food Inf..

[7] Radhakrishna R.B., Thomson J.S. (1996). Extension Agents’ Use of Information Sources. J. Ext..

[8] Strayer T.E., Kennedy L., Balis L., Ramalingam N., Wilson M., Harden S. (2020). Cooperative Extension Gets Moving, but How? Exploration of Extension Health Educators’ Sources and Channels for Information-Seeking Practices. Am. J. Health Promot..

[9] Luke D.A., Sarli C.C., Suiter A.M., Carothers B.J., Combs T.B., Allen J.L., Beers C.E., Evanoff B.A. (2018). The Translational Science Benefits Model: A New Framework for Assessing the Health and Societal Benefits of Clinical and Translational Sciences: Translational Science Benefits Model. Clin. Transl. Sci..

[10] Harden S., Washburn L., Berg A., Pena-Purcell N., Norman-Burgdolf H., Franz N. (2020). A Brief Report on a Facilitated Approach to Connect Cooperative Extension Southern Region State-Level Health Specialists. J. Hum. Sci. Ext..

[11] Balis L., Strayer T., Ramalingam N., Wilson M., Harden S. (2019). Open-Access Physical Activity Programs for Older Adults: A Pragmatic and Systematic Review. Gerontologist.

[12] Harden S.M., Ramalingam N., Breig S., Estabrooks P. (2019). Walk This Way: Our Perspectives on Challenges and Opportunities for Extension Statewide Walking Promotion Programs. J. Nutr. Educ. Behav..

[13] Aarons G., Sklar M., Mustanski B., Benbow N., Hendricks Brown C. (2017). “Scaling-out” Evidence-Based Interventions to New Populations or New Health Care Delivery Systems. Implement. Sci..

[14] Brownson R.C., Colditz G.A., Proctor E.K. (2017). Dissemination and Implementation Research in Health: Translating Science to Practice.

[15] Brownson R., Eyler A., Harris J., Moore J., Tabak R. (2018). Getting the Word Out: New Approaches for Disseminating Public Health Science. J. Public Health Manag. Pract..

[16] Lehoux P., Denis J.-L., Tailliez S., Hivon M. (2005). Dissemination of Health Technology Assessments: Identifying the Visions Guiding an Evolving Policy Innovation in Canada. J. Health Politics Policy Law.

[17] LSE Public Policy Group (2011). Maximizing the Impacts of Your Research: A Handbook for Social Scientists.

[18] Leeman J., Birken S.A., Powell B.J., Rohweder C., Shea C.M. (2017). Beyond “Implementation Strategies”: Classifying the Full Range of Strategies Used in Implementation Science and Practice. Implement. Sci..

[19] Damschroder L., Aron D., Keith R., Kirsh S., Alexander J., Lowery J. (2009). Fostering Implementation of Health Services Research Findings into Practice: A Consolidated Framework for Advancing Implementation Science. Implement. Sci..

[20] Powell B.J., Beidas R.S., Lewis C.C., Aarons G.A., McMillen J.C., Proctor E.K., Mandell D.S. (2017). Methods to Improve the Selection and Tailoring of Implementation Strategies. J. Behav. Health Serv. Res..

[21] Allen P., Sequeira S., Jacob R.R., Hino A.A.F., Stamatakis K.A., Harris J.K., Elliott L., Kerner J.F., Jones E., Dobbins M. (2013). Promoting State Health Department Evidence-Based Cancer and Chronic Disease Prevention: A Multi-Phase Dissemination Study with a Cluster Randomized Trial Component. Implement. Sci..

[22] Brownson R.C., Ballew P., Brown K.L., Elliott M.B., Haire-Joshu D., Heath G.W., Kreuter M.W. (2007). The Effect of Disseminating Evidence-Based Interventions That Promote Physical Activity to Health Departments. Am. J. Public Health.

[23] Cousins J.M., Langer S.M., Rhew L.K., Thomas C. (2011). The Role of State Health Departments in Supporting Community-Based Obesity Prevention. Prev. Chronic Dis..

[24] Davis M., Beidas R.S. (2021). Refining Contextual Inquiry to Maximize Generalizability and Accelerate the Implementation Process. Implement. Res. Pract..

[25] Davis M., Siegel J., Becker-Haimes E.M., Jager-Hyman S., Beidas R.S., Young J.F., Wislocki K., Futterer A., Mautone J.A., Buttenheim A.M. (2021). Identifying Common and Unique Barriers and Facilitators to Implementing Evidence-Based Practices for Suicide Prevention across Primary Care and Specialty Mental Health Settings. Arch. Suicide Res..

[26] Tabak R., Khoong E.C., Chambers D.A., Brownson R. (2012). Bridging Research and Practice Models for Dissemination and Implementation Research. Am. J. Prev. Med..

[27] Rabin B. Dissemination & Implementation Models in Health Research and Practice. https://dissemination-implementation.org/viewAll_di.aspx.

[28] Holt C.L., Chambers D.A. (2017). Opportunities and Challenges in Conducting Community-Engaged Dissemination/Implementation Research. Behav. Med. Pract. Policy Res..

[29] Damschroder L.J., Hagedorn H.J. (2011). A Guiding Framework and Approach for Implementation Research in Substance Use Disorders Treatment. Psychol. Addict. Behav..

[30] Rogers E. (2010). Diffusion of Innovations.

[31] Kumar V., Maheshwari B., Kumar U. (2002). Enterprise Resource Planning Systems Adoption Process: A Survey of Canadian Organizations. Int. J. Prod. Res..

[32] Creswell J., Plano Clark V. (2011). Designing and Conducting Mixed Methods Research.

[33] Castro F.G., Kellison J.G., Boyd S.J., Kopak A. (2010). A Methodology for Conducting Integrative Mixed Methods Research and Data Analyses. J. Mix. Methods Res..

[34] Harden S., Gunter K., Lindsay A. (2018). How to Leverage Your State’s Land Grant Extension System: Partnering to Promote Physical Activity. Transl. J. Am. Coll. Sport. Med..

[35] Kumar S., Phrommathed P. (2005). Research Methodology. New Product Development.

[36] Orcher L. (2016). Conducting Research.

[37] Downey S., Wages J., Jackson S.F., Estabrooks P.A. (2012). Adoption Decisions and Implementation of a Community-Based Physical Activity Program: A Mixed Methods Study. Health Promot. Pract..

[38] Harden S., Gaglio B., Shoup J., Kinney K., Johnson S., Brito F., Blackman K., Zoellner J., Hill J., Almeida F. (2015). Fidelity to and Comparative Results across Behavioral Interventions Evaluated through the RE-AIM Framework: A Systematic Review. Syst. Rev..

[39] Zoellner J., Krzeski E., Harden S., Cook E., Allen K., Estabrooks P.A. (2012). Qualitative Application of the Theory of Planned Behavior to Understand Beverage Consumption Behaviors among Adults. J. Acad. Nutr. Diet..

[40] Sullivan G.M., Artino A.R. (2013). Analyzing and Interpreting Data From Likert-Type Scales. J. Grad. Med. Educ..

[41] Polit D.F., Beck C.T. (2010). Generalization in Quantitative and Qualitative Research: Myths and Strategies. Int. J. Nurs. Stud..

[42] Hennink M., Kaiser B.N. (2022). Sample Sizes for Saturation in Qualitative Research: A Systematic Review of Empirical Tests. Soc. Sci. Med..

[43] Borkan J. (1999). Immersion/Crystallization. Doing Qualitative Research.

[44] Sparkes A.C., Smith B. (2013). Qualitative Research Methods in Sport, Exercise and Health.

[45] Strayer III T.E., Balis L.E., Kennedy L.E., Ramalingam N.S., Wilson M.L., Harden S.M. (2022). Intervention Characteristics Considered in Health Educators’ Adoption-Decision Making Process. Health Educ. Behav..

[46] Ensle K.M. (2005). Burnout: How Does Extension Balance Job and Family?. J. Ext..

[47] Kutilek L.M., Conklin N.L., Gunderson G. (2002). Investing in the Future: Addressing Work/Life Issues of Employees. J. Ext..

[48] Harden S., Steketee A., Glasgow T., Glasgow R., Estabrooks P. (2020). Suggestions for Advancing Pragmatic Solutions for Dissemination: Potential Updates to Evidence-Based Repositories. Am. J. Health Promot..

[49] Burkhardt J.T., Schröter D.C., Magura S., Means S.N., Coryn C.L.S. (2015). An Overview of Evidence-Based Program Registers (EBPRs) for Behavioral Health. Eval. Program Plan..

[50] Kennedy L.E., Strayer T.E.I., Balis L.E. (2022). Addressing Health Inequities: An Exploratory Assessment of Extension Educators’ Perceptions of Program Demand for Diverse Communities. Fam. Community Health.

[51] Balis L.E., Strayer T.E., Ramalingam N., Harden S.M. (2018). Beginning with the End in Mind: Contextual Considerations for Scaling-Out a Community-Based Intervention. Front. Public Health.

[52] Harden S., Balis L., Strayer T., Carlson B., Lindsay A., Dzewaltowski D., Estabrooks P., Gunter K. (2020). Strengths, Challenges, and Opportunities for Physical Activity Promotion in the Century-Old National Cooperative Extension System. J. Hum. Sci. Ext..

[53] Balis L.E., Gallup S., Norman-Burgdolf H., Buck J., Daniels P., Remley D., Graves L., Jenkins M., Price M. (2022). Unifying Multi-State Efforts Through a Nationally Coordinated Extension Diabetes Program. J. Hum. Sci. Ext..

